# Time-varing effect of policy uncertainty on A-share industry returns— A novel Bayesian approach

**DOI:** 10.1371/journal.pone.0326605

**Published:** 2025-06-25

**Authors:** Tianxing Zhu, Jinyang Liu, Daixing Zeng, Xuan Miao

**Affiliations:** 1 Jinan University-University of Birmingham Joint Institute, Jinan University, Guangzhou, China; 2 School of Statistics, Chengdu University of Information Technology, Chengdu, China; Universiti Malaysia Sabah, MALAYSIA

## Abstract

The impact of policy uncertainty on A-share industry returns shows significant time-varying characteristics, amplified by industry input-output relationships. Traditional TVP-VAR models overlook network structures, leading to unquantified spillover effects and imprecise systemic risk identification. To address this problem, this study embeds industry input-output tables as matrices into Time-Varying Parameter Spatial Autoregressive Model, and Bayesian methods are innovatively introduced into this model to capture the parameters. Policy uncertainty is categorized into five dimensions—economic, fiscal, monetary, exchange rate, and trade. Empirical results reveal following key findings: On average, network spillover effects explain approximately 39% of the response of A-share industry returns to policy uncertainty. Group analysis reveal that economic and fiscal policy uncertainties exhibit positive network effects, indicating synergistic effect that amplify their impact across industries. In contrast, exchange rate and trade policy uncertainties generate negative network effects, reflecting competitive or substitution effects. Systemic risk is most pronounced in fiscal and trade policy uncertainty groups. Systemic risk increases across all policy uncertainty groups except trade, which shows a declining trend. This study provides a novel framework for understanding the dual nature of spillover effect in production networks, offering valuable insights for policymakers and investors to manage systemic risks and indentify synergistic and competitive effects in interconnected industries.

## 1. Introduction

The Chinese economy has undergone remarkable growth over the past two decades. During this period, highly complex cross-sectional linkages were established between industries through input-output relationships, forming production networks whose existence has significant implications for the economic system. Specifically, production networks can amplify shocks in individual industries and propagate them to other industries through network connectivity, potentially triggering systemic risks [1,2]. Financial market volatility can affect various industries, generating impacts through different production dimensions and propagation paths [3–5]. Capturing systemic financial risk helps retrace past developments and provides forecasts and recommendations for the future.

However, with the rapid development of complex network theory, scholars have begun exploring the connectivity within the financial system and how these connections influence the propagation and amplification of risk [6]. The integration of spatial econometrics and Bayesian modeling offers an effective theoretical framework for analyzing the dynamics and spatial dependence of financial markets [7]. These methods enable more accurate modeling of spatial correlations and temporal variability among financial variables, offering richer insights for financial risk measurement and prediction.

Financial network spillovers – the propagation of risk events through interconnected institutions – fundamentally shape systemic risk dynamics. These spillovers amplify during crises, as distress at systemically important institutions propagates through trading linkages and market channels [8,9]. While financial networks typically stabilize market volatility, excessive connectivity can paradoxically amplify fluctuations and undermine this stabilizing function [10].

Recent studies have developed diverse methodologies for systemic risk measurement, with particular emphasis on tail risk spillovers during sector-wide extreme negative returns [11]. Building on this foundation, we advance a dynamic spatial Durbin model incorporating time-varying dependence parameters – a methodological innovation that enhances systemic risk quantification [12,13]. This framework provides unique insights into cross-industry risk transmission mechanisms.

Inter-sectoral changes are amplified into aggregate economic fluctuations through the transmission mechanism of production networks [14,15]. Input-output linkages are crucial in transmitting shocks [16]. However, some argue that heterogeneous shocks are dispersed by the law of large numbers in highly specialized economies, making it difficult to trigger aggregate economic fluctuations [17]. Subsequent studies have strongly refuted this view. Some scholars have noted that when the size distribution of firms exhibits thick tails, shocks to large firms cannot be offset by small firms, causing the Central Limit Theorem to fail, and micro shocks may trigger significant macroeconomic fluctuations [18].

Economic (EPU), fiscal (FP), monetary (MO), exchange rate (ER), and trade policy (TP) uncertainties significantly influence systemic financial risks and sectoral return volatility through production network transmission. EPU reduces firm investment efficiency, distorting industry yields and spillovers [19] (Al-Thaqeb & Algharabali, 2019), while FP amplifies network effects via sector-specific fiscal measures. Traditional panel models fail to capture MO’s time-varying dynamics [20], but someonedemonstrate its broad propagation using a Bayesian state-space network model [21]. ER and TP exacerbate transmission through global trade linkages and asymmetric network effects, respectively, with TP’s impact magnified by core sectors’ pivotal roles.

**Fig 1:**
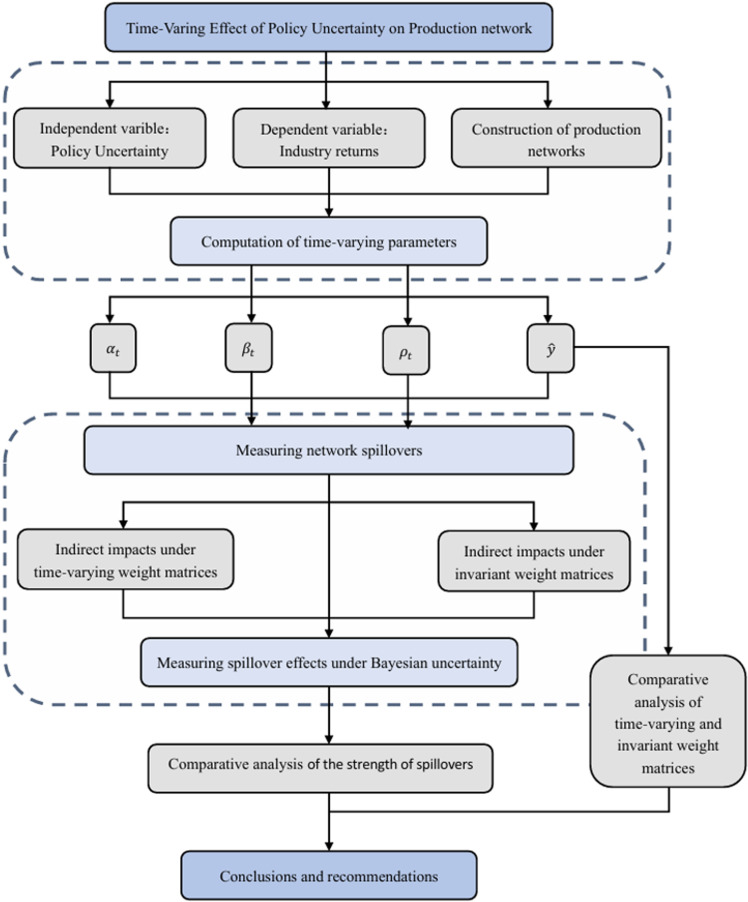
Flow chart.

This study develops a Bayesian Time-Varying Parameter Spatial Autoregressive (TVP-SAR) model with input-output-based spatial matrices to address the oversight of network structures in traditional models like TVP-VAR. The flow chart of this study is shown in Fig 1. Our objectives are: 1.To demonstrate the advantages of time-varying spatial matrices in capturing parameter uncertainty and risk dynamics; 2.To quantify the role of network effects in policy uncertainty transmission; 3.To identify distinct transmission channels—synergistic versus competitive.

Based on the existing theoretical and empirical literature, this study proposes the following two hypotheses: H1: The transmission of policy uncertainty is characterised by a synergistic network (neighbouring industries fluctuating in the same direction).H2: The transmission of policy uncertainty is characterised by a competitive network (core industries fluctuating inversely with peripheral industries).

Our framework advances related literature in three dimensions: (1) demonstrating time-varying matrices’ superiority in capturing parameter uncertainty and risk dynamics, (2) quantifying network effects’ role in policy transmission (accounting for 39% of A-share industry responses), and (3) identifying distinct transmission channels—synergistic (same-direction fluctuations among neighboring industries) versus competitive (inverse core-periphery fluctuations).

## 2. Research methodology

### 2.1. Bayesian TVP-SAR model

The Bayesian TVP-SAR model is defined as Eq (1):


$$Y_{it}=\rho_{t}W_{ijt}Y_{jt}+a_{t}+\beta_{it}X_{t}+\epsilon_{it},\epsilon_{it}\sim N(0,\sigma_{i}^{2})$$ (1)

Where $Y_{it}$ is the vector of industry returns of i at time t. $W_{ijt}$ is a pre-specified N × N weight matrix representing industry connections, which is a time-varying spatial weighting matrix constructed from input-output tables published by the National Statistical Office. $X_{t}$ is independent variable at time t. $\epsilon_{it}$ is a Gaussian error term with zero mean and variance $\sigma_{i}^{2}$. The network dependence structure is incorporated via $W_{t}$, where each element $W_{ijt}$ represents the relative importance of industry j to industry i at time t.

$\rho_{t}$ is a time-varying network dependence parameter that determines the strength of systemic risk [13], higher values of $\rho_{t}$ signify stronger systemic risk, suggesting that fluctuations in one industry are more likely to propagate to other industries. The spatial autoregressive coefficient $\rho_{t}$ captures risk spillovers by quantifying how shocks propagate through inter-industry networks. A positive $\rho_{t}$ indicates risk transmission, while a negative $\rho_{t}$ suggests a dampening effect. The term $\rho_{t}W_{t}$ explicitly models this propagation, where $W_{t}$ defines the strength of inter-industry connectivity. For example, a shock in industry i affects industry j through $\rho_{t}W_{t}$. The time-varying nature of $\rho_{t}$ allows for dynamic analysis of systemic risk, particularly during economic turbulence or policy uncertainty, when $\rho_{t}$ often increases, signaling stronger risk linkages.

A positive $\beta_{t}$ indicates that an increase in policy uncertainty leads to higher returns for the industry. This may occur in sectors that benefit from uncertainty, such as defensive industries (e.g., utilities or healthcare) or industries that thrive in volatile environments (e.g., commodities or speculative assets). A negative $\beta_{t}$ suggests that policy uncertainty reduces industry returns. This is common in sectors sensitive to economic stability, such as manufacturing or consumer goods, where uncertainty disrupts investment, production, or demand. A positive $\alpha_{t}$ indicates inherent profitability or resilience, reflecting a strong baseline performance in the absence of external shocks, while a negative $\alpha_{t}$ suggests structural challenges or inefficiencies, signaling vulnerability and potential reliance on external interventions to improve industry outcomes. This research assume the following random walk processes for the time-varying parameters $\rho_{t}$ as Eq (2)


$${\;\rho }_{t}=\rho_{t-1}+\varsigma \xi_{t},\xi_{t}\sim \;N(0,1)\;$$ (2)

Expressing Eq (1) in vector form yield Eq (3)


$$Y_{t}=\rho_{t}W_{t}Y_{t}+a+\beta_{t}X_{t}+\epsilon_{t}\;$$ (3)

In order to estimate the model Eqs (1) and (3), it is necessary to require that $I_{N}-\rho_{t}W_{t}$ is an invertible matrix for any t moments. Since $W_{t}$ is an asymmetric space-weighted matrix, $I_{N}-\rho_{t}W_{t}$ is invertible when ${\;\rho }_{t}$ is on (1,1/$r_{t,\min})$, where $r_{t,\min}$ is the largest of the negative real eigenvalues of $W_{t}$.

Bayesian TVP-SAR model represents a significant methodological advancement by incorporating three crucial features that address key limitations in existing literature: (1) The time-varying spatial parameter $\rho_{t}$ captures dynamic network spillover effects that traditional constant-parameter models cannot detect; (2) The input-output based spatial weight matrix $W_{t}$ explicitly accounts for inter-industry production linkages; (3) The Bayesian framework provides robust estimation of parameter uncertainty, particularly important for financial risk analysis where structural breaks are common. This innovative specification enables us to quantify how policy shocks propagate through China’s evolving production network while overcoming the model misspecification problems inherent in conventional approaches like TVP-VAR. The model’s superior performance in capturing risk transmission dynamics has been empirically validated through our comparative analysis.

### 2.2. Analysis of network effect

Morphing for the model Eqs (3) yields (4):


$$Y_{t}={\left(I_{N}-\rho_{t}W_{t}\right)}^{-1}\beta_{t}X_{t}+a+\epsilon_{t}$$ (4)

To analyze the effects of exogenous shocks, we derive the impact matrix as (5):


$$\frac{\partial Y_{t}}{\partial F_{t}}=S_{kt}={\left(I_{N}-\rho_{t}W_{t}\right)}^{-1}\beta_{t}$$ (5)

Where $\beta_{t}=diag(\beta_{1kt},\ldots ,\;\beta_{Nkt})$ contains the time-varying coefficients. ${\left(I_{N}-\rho_{t}W_{t}\right)}^{-1}$ represents the network propagation multiplier, capturing higher-order network effects. We decompose the impacts as: Direct impacts: Given by the diagonal elements of $S_{kt}$, representing the immediate impact of $F_{t}$ on each industry. Indirect impacts (network effects): Sum of off-diagonal elements, capturing spillovers across industries. Total impacts: Sum of all elements in a row, quantifying the overall impact.

A positive network effect indicates synergistic transmission, wherein policy uncertainty propagates across industries through industrial linkages (e.g., fiscal policy stimulating infrastructure-related sectors), information diffusion, and resource complementarity, thereby amplifying its overall impact. Conversely, a negative network effect represents competitive transmission, where policy uncertainty disseminates via resource competition (e.g., monetary policy redirecting capital to safe-haven industries), substitution dynamics (e.g., trade policy uncertainty incentivizing substitute industries), and risk dispersion, ultimately attenuating its influence. These distinct mechanisms underscore the heterogeneous spillover pathways of policy uncertainty across interconnected industries, emphasizing the need to differentiate between synergistic and competitive transmission channels in policy and investment analyses.

### 2.3. Estimation methods

Assuming that the network-dependent parameter $\rho_{t}$ is time-varying, increasing the difficulty of model estimation, the model Eqs (1) and (3) are estimated using Bayesian estimation. Where, for the initial state of the network dependent parameter $\rho_{0}$, a priori is chosen as (6)


$$\rho_{0}\sim N\left(\mu_{0},\sigma_{0}^{2}\right),\;\mu_{0}=0,\;\sigma_{0}^{2}=0.1$$ (6)

For the state new interest variance of the network-dependent parameters, a mildly informative inverse gamma prior is assumed as follows Eq (7)


$$\sigma_{i}^{2}\sim IG\left(c_{6},d_{\sigma }\right),c_{6}=3,d_{\sigma }=0.03$$ (7)

The log-likelihood function corresponding to Eq (3) is following Eq (8) [22]


$$L_{T}(\theta )=\;-\frac{NT}{2}\ln(2\pi )-\frac{T}{2}\sum_{i=1}^{N}\ln(\sigma_{i}^{2})+\frac{1}{2}\sum_{t=1}^{T}\ln|S_{t}S_{t}^{\prime }|-\frac{1}{2}\sum_{t=1}^{T}{\left[S_{t}Y_{t}-\beta_{t}X_{t}\right]}^{\prime }\times \Sigma^{-1}[S_{t}Y_{t}-\beta_{t}X_{t}]$$ (8)

Where $S_{t}=I_{N}-\rho_{t}W_{t}$, and $Y_{t}={\left(Y_{1t},Y_{2t},\ldots ,Y_{3t}\right)}^{\prime }$. Construct the posterior distribution as (9)


$$p(\Theta |Y)\propto [\prod_{t=1}^{T}p(Y_{t}|\rho_{t},b_{t}]\times p(\{\rho_{t}\},\{b_{t}\}|\sigma_{\rho }^{2},\Omega )\times p(\sigma_{\rho }^{2})$$ (9)

If $\rho_{t}$ and $b_{t}$ are both time-varying, it is also necessary to introduce a priori functions for the respective stochastic wandering processes; combined with the prior of $\sigma_{i}^{2}$ as $IG(a_{\sigma },b_{\sigma })$. The Gibbs + Metropolis-Hastings composite loop can be abbreviated as follows: 1. Update $\rho_{t}$: Given the other current parameters and data, do Metropolis-Hastings for each t or for the whole $\rho_{1:T}$, and compute the increment of the log-likelihood as (10)


$$\Delta (\rho^{⋆},\rho_{\text{old }})=\sum_{t=1}^{T}\ln p(Y_{t}|\rho^{⋆},b_{t})-\ln p(Y_{t}|\rho_{\text{old }},b_{t})+\ln p(\rho^{⋆})-\ln p(\rho_{\text{old }})$$ (10)

And accepted with probability as (11):


min{1,exp(Δ)} (11)

Likelihood increment: When $\rho_{t}$ is updated, the following (12) needs to be exponentiated before comparing it to 1.


$$\begin{array}{cc} \Delta (\rho^{⋆},\rho_{\text{old }}) & =\ln|I_{N}-\rho_{t}^{⋆}W_{t}|-\ln|I_{N}-\rho_{t}^{\text{(old) }}W_{t}|\\ & -\frac{1}{2}{\left(S_{t}^{⋆}Y_{t}-B_{t}X_{t}\right)}^{\prime }\Sigma^{-1}(S_{t}^{⋆}Y_{t}-B_{t}X_{t})\\ & +\frac{1}{2}{\left(S_{t}^{\text{(old) }}Y_{t}-B_{t}X_{t}\right)}^{\prime }\Sigma^{-1}(S_{t}^{\left(\text{old }\right)}Y_{t}-B_{t}X_{t})+\ln p(\rho_{t}^{⋆})-\ln p(\rho_{t}^{\left(\text{old }\right)}) \end{array}$$ (12)

Where $S_{t}^{⋆}$ satisfies (13)


$$S_{t}^{⋆}=I_{N}-\rho_{t}^{⋆}W_{t}$$ (13)

Updated $b_{t}$: If a Kalman filter is used to sample the stochastic wandering process, it is forward filtered, backward sampled, and then updated with the prior; in some pooling/shrinking cases, additional sampling is performed on the horseshoe prior or the mixture prior. Updated $\sigma_{i}^{2}$: given the residuals $\eta_{t,i}$, sampling from following (14):


$$\sigma_{i}^{2}\sim IG(a_{\sigma }+\frac{T}{2},b_{\sigma }+\frac{1}{2}\sum_{t=1}^{T}\eta_{t,i}^{2})$$ (14)

Update $\sigma_{i}^{2}$: Similarly the inverse gamma prior can be applied to the error variance $\sigma_{\rho }^{2}$ of $\rho_{t}$, combining $\rho_{t}$-$\rho_{t-1}$ for sum-of-squares sampling.

Expansion of the Mixture and shrink a priori formulas: Horseshoe a priori (shrink). For each coefficient $\beta_{i}$ or $\beta_{i,t}$ assigngiven by (15)


$$\beta_{i}\sim N\left(0,\tau^{2}\lambda_{i}^{2}\right),\lambda_{i}^{2}\sim C^{+}(0,1),\tau \sim C^{+}(0,1)$$ (15)

Mixture A priori (mix): Let $\beta_{i}$ belong to a certain grouping g∈{1,...,G} that satisfies (16)


$$p\left(s_{i}=g\right)=k_{g},\sum_{g=1}^{G}k_{g}=1$$ (16)

And on each group following (17) is satisfied:


$$\;\beta_{i}|s_{i}=g\sim N(\mu_{g},V_{g})$$ (17)

After completing the MCMC, in order to evaluate the model fitting and prediction effect, the following operations are usually done: a posteriori mean or median parameter: take the mean/median on the MCMC sample for $\rho_{t}$,βi,t,$\sigma_{i}^{2}$ to get the a posteriori estimate. Generate predicted values: as follows (18)


Y^t=(IN−ρ^tWt)−1∑i=1N[β^i,tXi,t] (18)

Thus (19) can be derived as follows.


yhatt=(IN−ρt, post (mean )Wt)−1(∑kb^k,t·Xk,t+ϵt) (19)

and use it to compare with the real $Y_{t}$. RMSE is given by (20)


RMSEi=1T∑t=1T(Yi,t−Y^i,t)2 ,RMSEtotal =1NT∑i=1N∑t=1T(Yi,t−Y^i,t)2(Yi,t−Y^i,t)2 (20)

**Fig 2:**
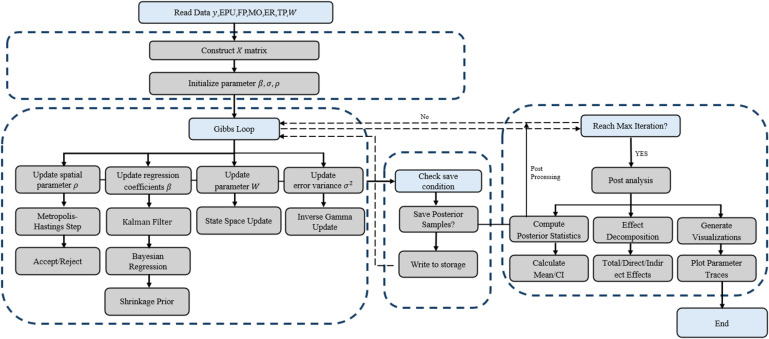
Flow chart of algorithm.

It can also be displayed separately by industry or aggregated into a total measure. Posterior confidence intervals: Generate intervals by taking, e.g., 16%−84%, 5%−95% quartiles. The code get.post is doing something similar to measure uncertainty. The flow chart of algorithm is shown in Fig 2. The algorithm table is shown as follows:

**Algorithm 1** Bayesian Dynamic Spatial Panel Model Estimation Framework

**Input**

Dataset of interest: returns, policy uncertainty index, spatial weight matrices over time.

Model configuration: time-varying parameters (TVP), spatial dependence type (none, constant, or TVP), pooling strategy (free, shrink, mixture)

Hyperparameters: number of MCMC iterations $N_{tot}$, burn-in $N_{burn}$, thinning interval, prior variances, etc.

Optional: shrinkage priors (horseshoe), mixture components G, identification via permutation sampling

**Output**

Posterior draws of coefficients $\alpha_{t},\beta_{t},$ variances ∑, spatial coefficients $\rho_{t}$, group indicators $s_{i}$.

**Main loop**

**for** iteration=1 to $N_{tot}$ **do**

1.Prepare Data

 a.Select spatial weight matrix $W_{t}$ based on time.

 b.Apply spatial transformation if $\rho_{t}\neq 0,\;y^{*}=(I_{N}-\rho_{t}W_{t})y$

2.Sample Model Parameters

 a. If pooling strategy is free or shrink, draw individual coefficients $\alpha_{t},\beta_{t}$

 b. If TVP is enabled, sample time-varying $\beta_{i,t}$via Kalman filtering

 c. If shrinkage is enabled, update hyperparametersλ,ν,τ,ζ

 d. If mixture prior is enabled, update group indicators $s_{i}$ and group means $\mu_{g}$

3.Sample Error Variance Parameters

 a. If heteroskedastic, draw $\sigma_{i}^{2}$ for each unit

 b. If grouped, update group-specific variances

4.Sample Spatial Autoregressive Parameter $\rho_{t}$

 a. For constant or time-varying ρ, use Metropolis-Hastings

 b. Adapt step size to maintain target acceptance

5.Store Posterior Draws

 a.Save sampled $\alpha_{t},\beta_{t},\;{\rho_{t},\mu }_{g},s_{i}$

 b.Optionally, compute and store fitted values $y_{hatt}$

Post-processing and Output

1. Compute posterior summaries (mean, credible intervals)

2. Evaluate predictive accuracy: $RMSE=\sqrt{\Sigma {\left(y-\hat{y}\right)}^{2}/TN}$

3. Estimate structural impacts (direct, indirect, total effects) of covariates.

4. Export results to output files: posterior draws, impact tables, fitted values

### 2.4. Data sources and processing

This study uses the China Securities Index 500 to obtain monthly industry returns as the dependent variable, which covers the period from August 2015 to the present. The weight matrix is constructed based on the input-output tables of non-competitive industries published by the China Bureau of Statistics in 2012 and 2017. Due to model limitations, this study only numerically compares the regression results of time-varying power recombination (W) and constant power recombination (Wct)

Based on the authoritative classification framework developed by the Centre for Digital Economics at Hong Kong Baptist University, this study adopts a systematic and objective approach to policy uncertainty by dividing it into five key dimensions: the Economic Policy Uncertainty Index (EPU), Fiscal Policy Uncertainty Index (FP), Monetary Policy Uncertainty Index (MO), Exchange Rate Policy Uncertainty Index (ER), and Trade Policy Uncertainty Index (TP). This multi-dimensional classification enhances the study’s comprehensiveness and scientific rigor while minimizing potential subjective bias. The five indices cover the period from January 2000 to May 2022. Combined with the timing of the data for the dependent variable, this study finalised the sample observation period from August 2015 to May 2022.

Regarding data processing, to improve the interpretability of regression coefficients and prevent excessively small parameter estimates, all policy uncertainty indices (EPU, FP, MO, ER, and TP) were standardized by dividing their raw values by 1,000. This adjustment preserves the relative relationships between the variables while keeping the regression coefficients from being too large or too small, ensuring that the regression results are clearer and more intuitive, in line with standard econometric practice.

The CSI 500 index selection follows a rigorous three-step process: excluding CSI 300 constituents and top 300 stocks by market cap, removing the least liquid 20%, then selecting the top 500 remaining stocks by market cap. Industry classification adheres to CSRC’s 2012 standards (18 industries, excluding composites; see Appendix Table A1), ensuring representative mid-cap coverage while maintaining methodological consistency with Chinese market research practices.

**Fig 3:**
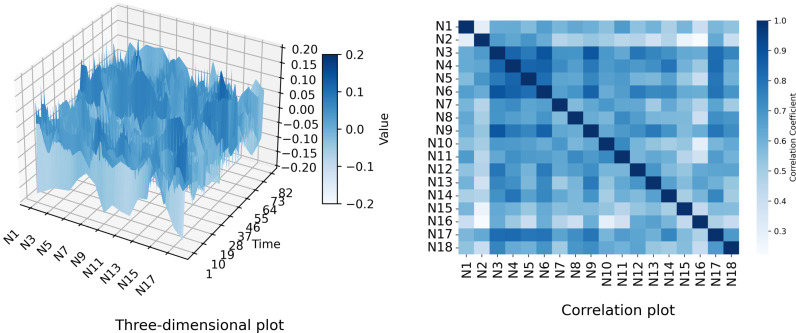
Dependent variable.

**Fig 4:**
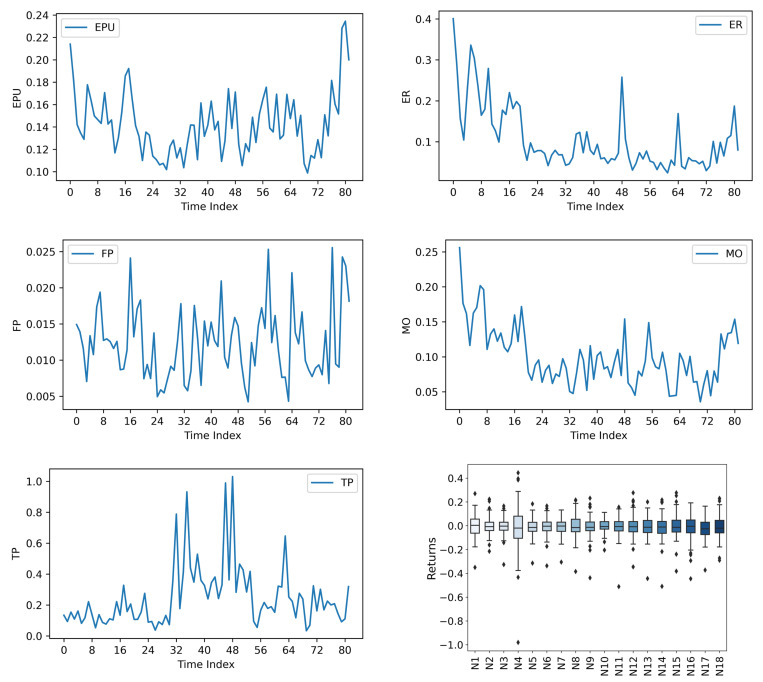
Independent variable.

Descriptive statistics of the sample are presented in Table 1. Fig 3 below illustrates the dependent variable in the form of a three-dimensional plot and a correlation plot, respectively. Fig 4 below demonstrates the independent variable in the form of a line graph and a box plot:

**Table 1:** Descriptive statistics.

	mean	std	min	max
EPU	142.75	28.47	98.87	234.52
FP	12.33	5.05	4.23	25.56
MO	100.54	41.60	35.71	256.03
ER	105.72	78.17	24.07	400.53
TP	247.95	200.41	33.41	1031.01

## 3. Empirical analysis

### 3.1. Regression results of $\beta_{t}$ and $\alpha_{t}$

**Fig 5:**
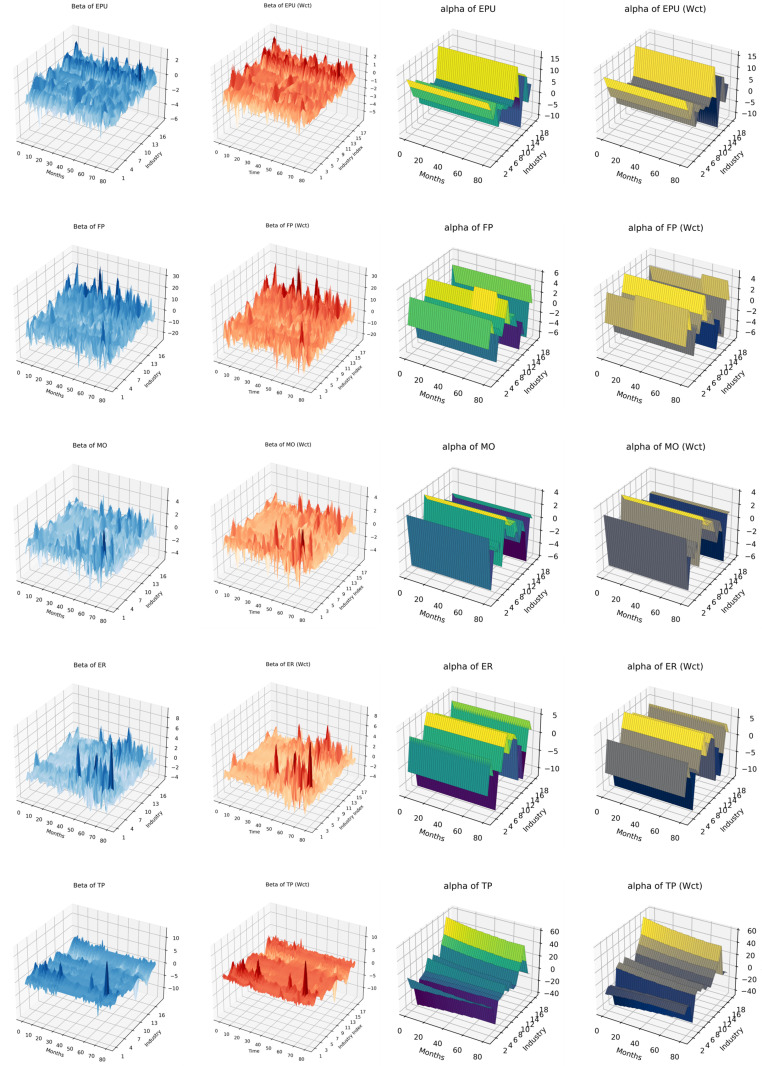
*α* and *β.*

A positive $\beta_{t}$ indicates that an increase in policy uncertainty leads to higher returns for the industry. This may occur in sectors that benefit from uncertainty, such as defensive industries (e.g., utilities or healthcare) or industries that thrive in volatile environments (e.g., commodities or speculative assets). A negative $\beta_{t}$ suggests that policy uncertainty reduces industry returns. This is common in sectors sensitive to economic stability, such as manufacturing or consumer goods, where uncertainty disrupts investment, production, or demand. A positive $\alpha_{t}$ indicates inherent profitability or resilience, reflecting a strong baseline performance in the absence of external shocks, while a negative $\alpha_{t}$ suggests structural challenges or inefficiencies, signaling vulnerability and potential reliance on external interventions to improve industry outcomes. Through model iteration of 15000 times, this study obtains the monthly $\beta_{t}$ as well as $\alpha_{t}$ in Fig 5. The images labeled as Wct are processed using an invariant weight matrix, whereas the others are processed using a time-varying weight matrix group.

$\beta_{t}$ analysis: under EPU, FP, an MO, $\beta_{t}$ estimates exhibit significant volatility across industries, reflecting the broad and unpredictable impact of these policies on market confidence and sectoral performance. In contrast, ER group presents a more heterogeneous pattern, with industry-specific differences in beta fluctuations. Notably, Transportation Equipment (N7) experiences the largest fluctuations in beta under exchange rate policy uncertainty (ER), highlighting its strong dependence on exchange rate stability, as currency fluctuations directly influence trade costs and supply chain reliability. TP results in relatively lower beta volatility across industries, suggesting that while trade policy changes impact economic sectors, the response remains more stable compared to other policy uncertainties.

$\alpha_{t}$ analysis: under FP, MO, ER, indicating that these policy shifts significantly affect industry profitability. In contrast, EPU and TP also present large variations in baseline alpha values, suggesting that even sectors typically considered stable are impacted by these uncertainties. Within TP, Resident Services (N15) exhibits a notably high alpha baseline, reflecting the inelastic demand for accommodation-related services, which remain essential despite trade fluctuations. Meanwhile, under FP, Education (N16) shows the lowest alpha baseline, indicating its vulnerability to government budgetary uncertainty, as reductions in public funding directly weaken the sector’s financial stability.

Comparison between the time-varying weight matrix and the fixed weight matrix (Wct) reveals that the Wct model results in more pronounced beta fluctuations, while the overall trend remains consistent across both approaches. This suggests that assuming static industry relationships amplifies short-term volatility, whereas the general model better captures adaptive market responses. In contrast, alpha values show minimal differences between the two models, indicating that long-term industry profitability remains largely stable regardless of whether industry linkages are assumed to be dynamic or fixed.

**Fig 6:**
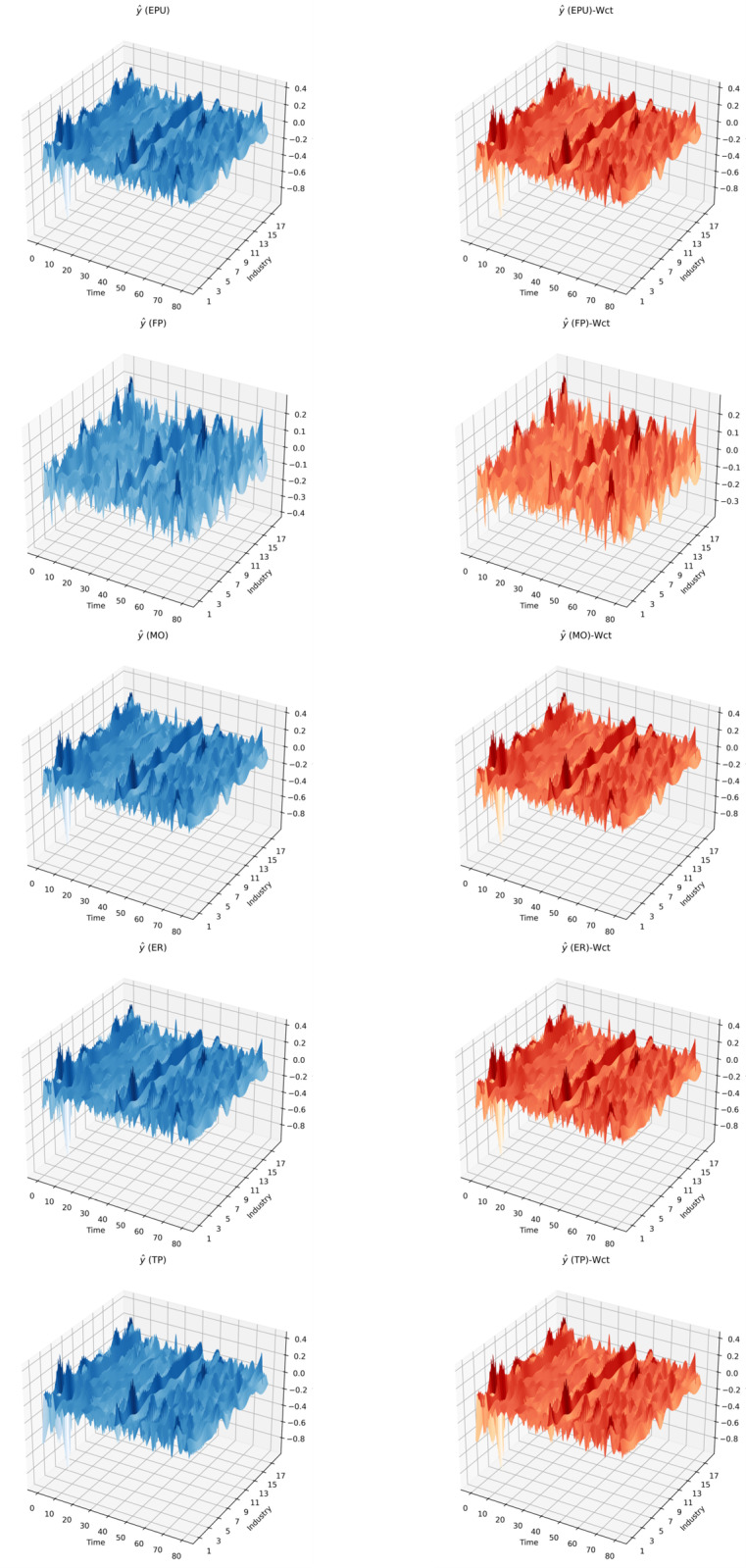
$\hat{y}$.

The yhat obtained in this study based on Bayes’ Rule is shown below in Fig 6:

### 3.2. Production network construction

**Fig 7:**
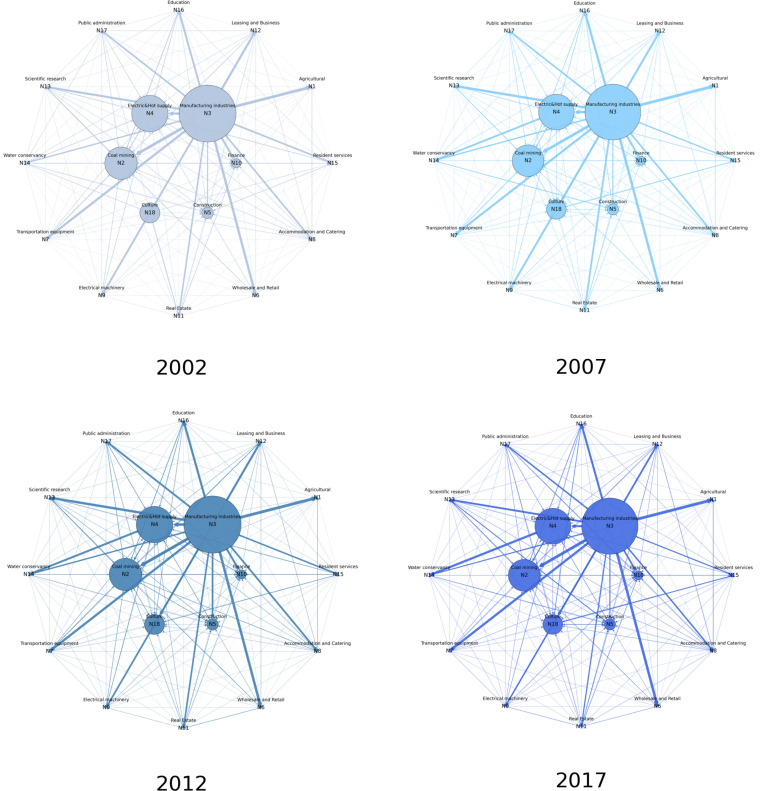
Production network construction.

Utilizing industry input-output tables, this study calculates the net inflows and outflows for each industry. Based on these results, we construct a production network diagram that distinguishes node sizes and arrow directions to represent the magnitude and direction of flows. The results are illustrated in Fig 7. This study shows the last four industry input-output tables published by the National Bureau of Statistics of China for 2002, 2007, 2012 and 2017. This visualization captures the temporal evolution of uncertainty transmission across industries, highlighting the structural shifts in inter-industry relationships over time.

The sustainable production network reveals the directional input and ouput relationship across industries, highlighting the key drivers of China’s economic structure. In the first half of the sample period, industries such as manufacturing (N3), electricity, heat, gas, and water production and supply (N4), and mining (N2) exhibit high values, indicating their dominant role in transmitting uncertainty to other sectors. Service industries like culture, sports, and entertainment (N18) and financial services (N10) also show significant uncertainty spillovers, reflecting their growing influence on the economy. In the second half of the period, the production network captures the rising importance of leasing and business services (N12) and construction (N5), with strong directional flows from traditional industries to these modern sectors.

The high values between manufacturing and service industries underscore the transition from an industry-led economy to a more balanced industrial-service structure. Notably, the leasing and business services industry (N12) not only receives substantial uncertainty spillovers from manufacturing but also transmits uncertainty to other service sectors, highlighting its role in enhancing efficiency and reducing costs. Similarly, the culture, sports, and entertainment industry (N18) demonstrates strong bidirectional uncertainty flows, reflecting its integration into both traditional and modern economic frameworks.

Overall, the production network provides a robust framework for analyzing the directional input and output relationship across industries. It confirms the foundational role of manufacturing while emphasizing the increasing importance of service industries in driving economic transformation. This shift is clearly captured by the production network, offering valuable insights for optimizing industrial policies and promoting high-quality development.

### 3.3. Systemic risk factors $\rho_{t}$

**Fig 8:**
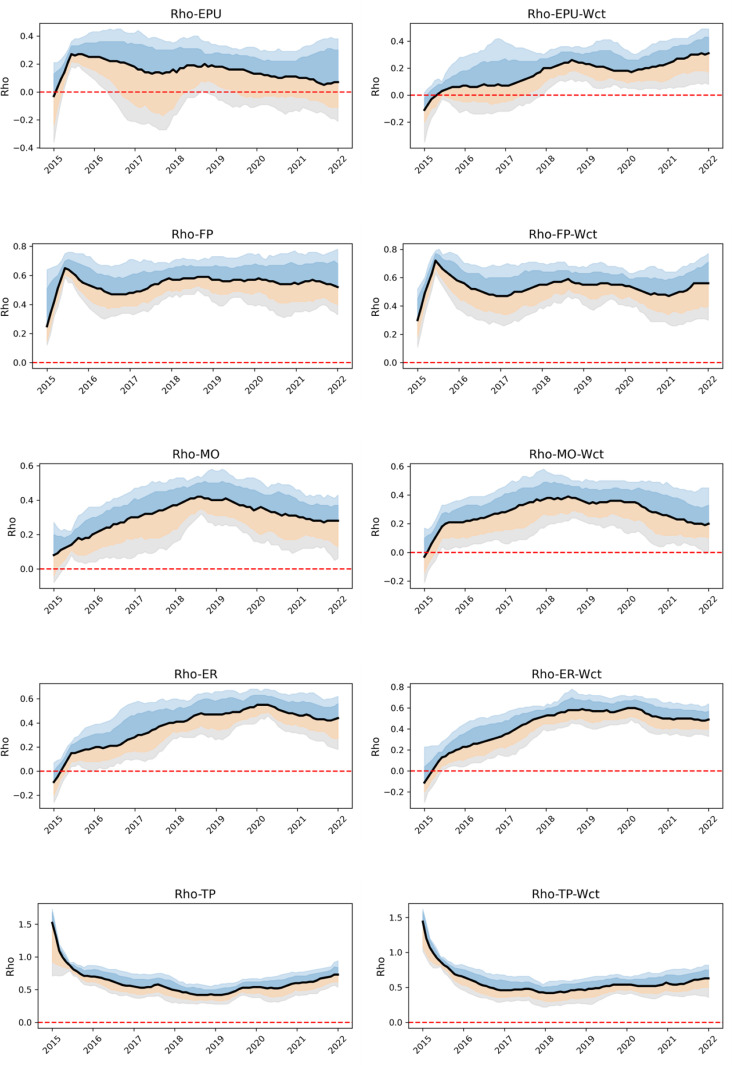
$\rho_{t}$.

Table 2 shows $\rho_{t}$ of both time-varying weight matrix and invariant weight matrix group, and the value of systemic risk factors were plotted in Fig 8, for each of the two interval values and one value inside the table, the first interval indicates the 16% to 84% confidence interval, the second interval indicates the 5% to 95% confidence interval, and the value above the two interval indicates the mean value.

**Table 2:** $\rho_{t}$ (annual average).

W	EPU	FP	MO	ER	TP
2015	0.09 (−0.03,0.19) (−0.10,0.24)	0.42 (0.34,0.58) (0.30,0.66)	0.11 (0.03,0.19) (−0.02,0.24)	0.00 (−0.08,0.08) (−0.13,0.13)	1.17 (0.87,1.26) (0.72,1.30)
2016	0.25 (0.16,0.35) (0.10,0.41)	0.56 (0.48,0.66) (0.43,0.73)	0.20 (0.11,0.28) (0.05,0.34)	0.18 (0.09,0.28) (0.03,0.36)	0.73 (0.64,0.81) (0.59,0.87)
2017	0.18 (−0.06,0.34) (−0.18,0.42)	0.48 (0.39,0.60) (0.34,0.69)	0.29 (0.17,0.39) (0.08,0.46)	0.27 (0.15,0.44) (0.08,0.54)	0.56 (0.40,0.61) (0.33,0.67)
2018	0.16 (−0.08,0.31) (−0.16,0.38)	0.56 (0.48,0.65) (0.44,0.70)	0.37 (0.24,0.47) (0.17,0.52)	0.40 (0.30,0.52) (0.24,0.59)	0.50 (0.40,0.61) (0.33,0.67)
2019	0.18 (0.04,0.28) (−0.05,0.34)	0.58 (0.49,0.66) (0.44,0.72)	0.40 (0.32,0.50) (0.27,0.56)	0.48 (0.36,0.59) (0.30,0.66)	0.43 (0.35,0.52) (0.30,0.58)
2020	0.13 (−0.03,0.24) (−0.10,0.30)	0.57 (0.47,0.67) (0.40,0.74)	0.35 (0.23,0.45) (0.17,0.51)	0.53 (0.45,0.61) (0.41,0.67)	0.53 (0.43,0.62) (0.36,0.67)
2021	0.10 (−0.04,0.25)(−0.14,0.32)	0.55 (0.42,0.68) (0.35,0.75)	0.30 (0.18,0.40) (0.11,0.45)	0.46 (0.37,0.57) (0.30,0.64)	0.60 (0.48,0.71) (0.41,0.77)
2022	0.06 (−0.10,0.30) (−0.18,0.38)	0.54 (0.42,0.69) (0.37,0.76)	0.28 (0.15,0.37) (0.08,0.42)	0.43 (0.29,0.54) (0.21,0.61)	0.71 (0.60,0.81) (0.54,0.88)
Wct	EPU	FP	MO	ER	TP
2015	−0.04 (−0.12,0.03)(−0.19,0.07)	0.47 (0.39,0.57)(0.32,0.62)	0.06 (−0.04,0.15)(−0.10,0.20)	−0.01 (−0.10,0.11)(−0.16,0.24)	1.12 (0.92,1.25)(0.87,1.28)
2016	0.06 (−0.04,0.17)(−0.10,0.25)	0.60 (0.50,0.69)(0.43,0.74)	0.21 (0.11,0.31)(0.04,0.36)	0.21 (0.11,0.33)(0.05,0.41)	0.69 (0.59,0.78)(0.53,0.83)
2017	0.08 (−0.04,0.25)(−0.11,0.38)	0.48 (0.36,0.60)(0.28,0.68)	0.28 (0.15,0.39)(0.08,0.46)	0.35 (0.23,0.48)(0.15,0.55)	0.48 (0.37,0.60)(0.32,0.68)
2018	0.18 (0.09,0.28)(0.03,0.33)	0.54 (0.44,0.64)(0.38,0.70)	0.37 (0.24,0.47)(0.17,0.55)	0.52 (0.43,0.60)(0.36,0.64)	0.44 (0.33,0.54)(0.26,0.60)
2019	0.23 (0.14,0.32)(0.08,0.37)	0.56 (0.48,0.64)(0.42,0.69)	0.36 (0.26,0.45)(0.21,0.50)	0.58 (0.48,0.67)(0.43,0.73)	0.48 (0.36,0.58)(0.29,0.63)
2020	0.18 (0.11,0.28)(0.05,0.35)	0.53 (0.45,0.61)(0.40,0.66)	0.34 (0.23,0.44)(0.14,0.50)	0.58 (0.49,0.66)(0.44,0.71)	0.53 (0.45,0.62)(0.39,0.68)
2021	0.25 (0.13,0.35)(0.06,0.41)	0.49 (0.36,0.60)(0.28,0.66)	0.25 (0.12,0.38)(0.06,0.46)	0.5 (0.41,0.60)(0.35,0.65)	0.55 (0.44,0.67)(0.38,0.74)
2022	0.30 (0.18,0.42)(0.08,0.48)	0.56 (0.39,0.68)(0.31,0.74)	0.2 (0.11,0.32)(0.02,0.44)	0.49 (0.40,0.57)(0.34,0.63)	0.62 (0.49,0.73)(0.38,0.80)

As shown in Table 2 and Fig 8, the overall trend analysis reveals that multiple groups of systemic risk exhibit a gradual upward trajectory under both time-varying and non-varying weight restructuring. The annual mean values of the variables show instability, reflecting the significant impact of specific economic events. Specifically, the EPU and FP groups demonstrate a surge followed by smoother fluctuations, MO and ER groups show a slow increase followed by a gradual decrease, the TP group exhibits a decrease followed by stable fluctuations. Both the time-varying weights group and the invariant weights matrix group display similar changes in mean values, though variability in the confidence intervals is observed.

The EPU group shows lower mean values with limited fluctuations over time and the widest confidence intervals, indicating the relatively stable systemic risk of industries, a slight increase is observed between 2016 and 2017, likely due to the early stages of the U.S.-China trade friction and global economic policy adjustments, but this flattens out after 2020 [23]. The FP group, by contrast, has a higher mean value, peaking significantly between 2016 and 2020. This reflects the substantial risk spillover between industries, such as global fiscal expansion during the epidemic. The value systemic risk factor of MO group is relative low. The ER group’s mean increases significantly, peaking in 2018 with value 0.54. The TP group shows a higher mean, which gradually declines from 2015 to 2022, possibly due to the reduction of trade policy uncertainty on industries in the post-epidemic period.

Our comparative analysis reveals important economic insights about risk transmission dynamics. While significant mean differences between time-varying and invariant weights appear primarily in the EPU group, the consistently wider confidence intervals under time-varying weights across all five policy groups (EPU, FP, MO, ER, TP) carry deeper implications. This pattern reflects fundamental economic realities: (1) The financial system’s evolving network structure creates time-dependent transmission channels – during periods of market stress or policy shifts, certain industries become disproportionately exposed to specific risks; (2) Industry specialization means heterogeneous responses to policy shocks (e.g., trade-sensitive sectors react more strongly to TP shocks); (3) The invariant weights’ narrower bands essentially average out these crucial temporal and cross-sectional variations, potentially masking early warning signals of systemic risk buildup. Our findings align with the volatility paradox concept, where seemingly stable averages conceal growing underlying vulnerabilities – precisely what our time-varying approach helps uncover. This has critical policy implications: regulators monitoring average spillovers might miss accumulating sector-specific risks that our method detects through wider confidence bands.

### 3.4. Direct, indirect (spillover) and total impacts

**Fig 9:**
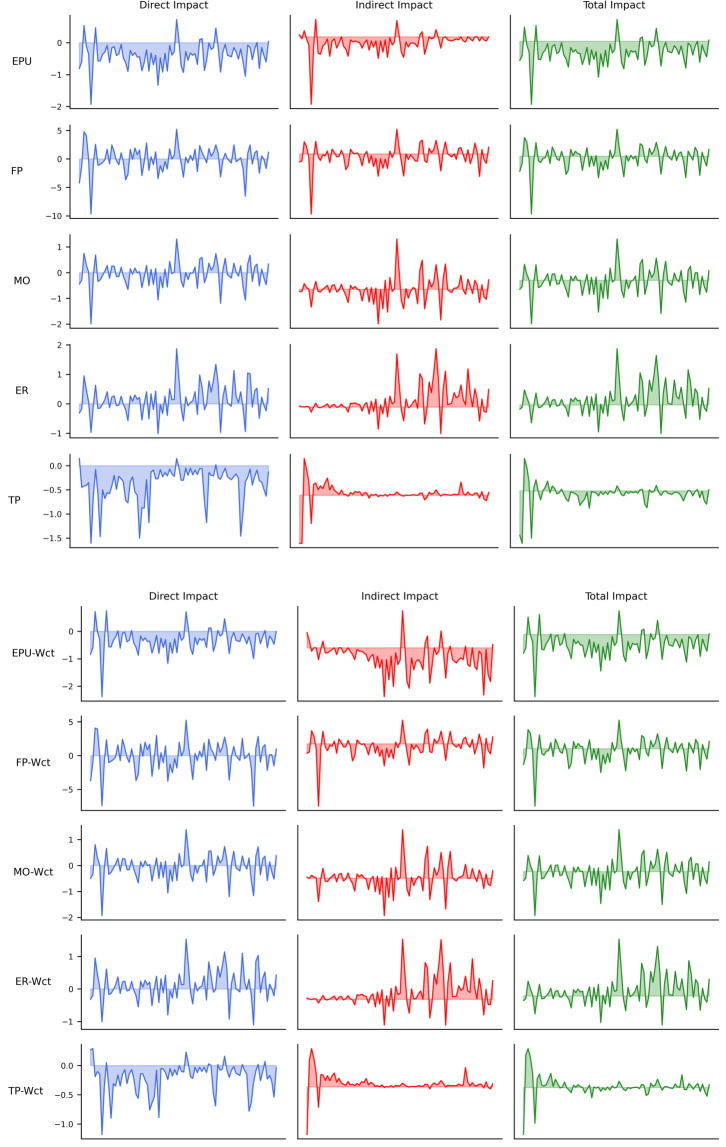
Time-varying graphs of mean impact.

**Fig 10:**
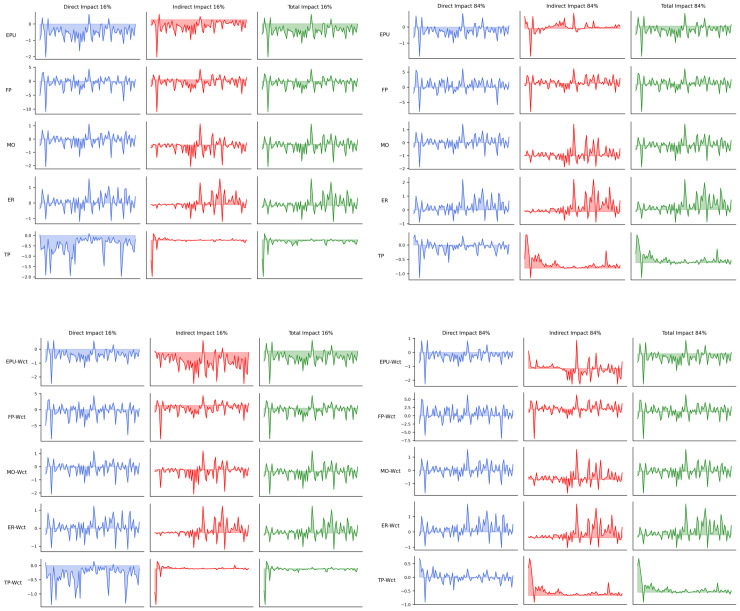
Impact under confidence intervals.

Table 3 shows the direct (Dir), indirect (Ind), and total impacts (Tot) of time-varying (W) and non-varying power reorganization (Wct). Table 4 shows the mean impact of all groups, Ave denotes the average value of five groups. Fig 9 shows the mean value over time. Fig 10 shows the value over time under confidence intervals.

**Table 3:** Impacts.

W	EPU			FP		
	Dir	Ind	Tot	Dir	Ind	Tot
2015	−0.26 (−0.40,-0.13) (−0.49,-0.07)	−0.01 (−0.09,0.08) (−0.14,0.12)	−0.28 (−0.4,-0.15) (−0.47,-0.1)	0.67 (−0.32,1.71) (−0.89,2.40)	0.03 (−0.76,0.75) (−1.36,1.27)	0.7 (−0.36,1.72) (−1.21,2.61)
2016	−0.42 (−0.54,-0.31) (−0.60,-0.25)	−0.12 (−0.20,-0.06) (−0.26,-0.03)	−0.55 (−0.7,-0.41) (−0.77,-0.34)	−0.56 (−1.13,0.03) (−1.49,0.45)	−1.21 (−1.89,-0.52) (−2.43,0.11)	−1.77 (−2.79,-0.69) (−3.56,0.22)
2017	−0.48 (−0.62,-0.33) (−0.70,-0.27)	−0.07 (−0.17,0.04) (−0.25,0.10)	−0.54 (−0.68,-0.42) (−0.78,-0.34)	−0.37 (−0.97,0.30) (−1.36,0.72)	−0.68 (−1.36,0.00) (−1.96,0.61)	−1.06 (−2.08,0.07) (−2.88,0.93)
2018	−0.58 (−0.75,-0.40) (−0.82,-0.33)	−0.09 (−0.20,0.06) (−0.26,0.11)	−0.65 (−0.76,-0.53) (−0.84,-0.47)	−0.5 (−1.12,0.16) (−1.51,0.57)	−1.59 (−2.29,-0.92) (−2.85,-0.35)	−2.14 (−3.15,-0.98) (−3.96,-0.05)
2019	−0.3 (−0.43,-0.17) (−0.49,-0.10)	−0.05 (−0.12,0.02) (−0.17,0.07)	−0.34 (−0.47,-0.22) (−0.56,-0.15)	0.46 (−0.10,1.06) (−0.46,1.43)	−0.16 (−0.88,0.57) (−1.44,1.15)	0.28 (−0.73,1.40) (−1.55,2.26)
2020	−0.25 (−0.37,-0.13) (−0.43,-0.08)	−0.03 (−0.08,0.04) (−0.12,0.07)	−0.27 (−0.39,-0.17) (−0.46,-0.11)	0.44 (−0.18,1.10) (−0.63,1.48)	0.4 (−0.19,1.09) (−0.65,1.70)	0.87 (−0.14,1.92) (−0.89,2.76)
2021	−0.35 (−0.48,-0.23) (−0.54,-0.17)	−0.02 (−0.07,0.02) (−0.11,0.05)	−0.37 (−0.5,-0.26) (−0.58,-0.20)	−0.16 (−0.64,0.36) (−0.95,0.69)	−0.02 (−0.68,0.71) (−1.23,1.40)	−0.18 (−1.12,0.88) (−1.88,1.76)
2022	−0.38 (−0.50,-0.25) (−0.55,-0.19)	−0.02 (−0.12,0.05) (−0.17,0.09)	−0.4 (−0.49,-0.34) (−0.54,-0.29)	−0.5 (−1.17,0.31) (−1.55,0.70)	−1.15 (−1.90,-0.51) (−2.44,-0.01)	−1.68 (−2.44,-0.82) (−3.05,-0.04)
W	MO			ER		
	Dir	Ind	Tot	Dir	Ind	Tot
2015	0.07 (0.02,0.12) (−0.01,0.15)	0.01 (−0.03,0.05) (−0.06,0.07)	0.08 (0.05,0.10) (0.03,0.12)	0.2 (0.16,0.24) (0.13,0.26)	0.01 (−0.02,0.05) (−0.04,0.07)	0.21 (0.19,0.24) (0.17,0.26)
2016	−0.14 (−0.18,-0.09) (−0.21,-0.06)	−0.01 (−0.05,0.02) (−0.07,0.04)	−0.15 (−0.19,-0.11) (−0.21,-0.09)	0.02 (−0.03,0.05) (−0.05,0.07)	0.02 (−0.01,0.05) (−0.03,0.07)	0.03 (−0.00,0.06) (−0.02,0.08)
2017	−0.13 (−0.19,-0.08) (−0.23,-0.04)	−0.03 (−0.07,0.00) (−0.1,0.03)	−0.17 (−0.23,-0.11) (−0.26,-0.06)	−0.01 (−0.07,0.06) (−0.12,0.10)	0.03 (−0.01,0.10) (−0.04,0.15)	0.03 (−0.05,0.11) (−0.09,0.18)
2018	−0.18 (−0.28,-0.11) (−0.34,-0.06)	−0.12 (−0.18,-0.04) (−0.22,0.01)	−0.31 (−0.38,-0.23) (−0.43,-0.17)	−0.03 (−0.12,0.05) (−0.17,0.12)	−0.02 (−0.09,0.06) (−0.13,0.12)	−0.05 (−0.15,0.06) (−0.21,0.13)
2019	0.07 (−0.00,0.14) (−0.04,0.19)	0.05 (−0.01,0.12) (−0.06,0.16)	0.12 (0.05,0.20) (−0.01,0.25)	0.3 (0.19,0.42) (0.12,0.49)	0.31 (0.20,0.46) (0.13,0.56)	0.62 (0.50,0.76) (0.42,0.86)
2020	0.09 (−0.01,0.18) (−0.07,0.24)	0.08 (−0.0,0.17)(−0.05,0.22)	0.17 (0.10,0.25) (0.05,0.30)	0.35 (0.23,0.47) (0.15,0.55)	0.51 (0.37,0.67) (0.28,0.79)	0.86 (0.71,1.03) (0.62,1.16)
2021	−0.0 (−0.07,0.06) (−0.11,0.10)	0.05 (0.0,0.09)(−0.02,0.12)	0.04 (−0.03,0.12) (−0.08,0.17)	0.3 (0.21,0.39) (0.15,0.45)	0.38 (0.26,0.57) (0.19,0.76)	0.69 (0.52,0.90) (0.44,1.13)
2022	−0.14 (−0.21,-0.07) (−0.26,-0.04)	−0.06 (−0.12,0.01) (−0.16,0.05)	−0.2 (−0.24,-0.16) (−0.26,-0.13)	−0.0 (−0.10,0.10) (−0.16,0.18)	0.01 (−0.08,0.10) (−0.13,0.19)	0.0 (−0.08,0.10) (−0.12,0.22)
Wct	EPU			FP		
	Dir	Ind	Tot	Dir	Ind	Tot
2015	0.19 (0.14,0.24) (0.10,0.28)	0.02 (−0.02,0.07) (−0.05,0.11)	−0.18 (−0.26,-0.09) (−0.32,-0.04)	0.66 (−0.19,1.45) (−0.72,2.01)	−0.06 (−0.83,0.72) (−1.38,1.22)	0.70 (−0.36,1.72) (−1.21,2.61)
2016	−0.00 (−0.04,0.04) (−0.06,0.07)	−0.02 (−0.07,0.02) (−0.13,0.05)	−0.42 (−0.51,-0.33) (−0.58,-0.27)	−0.37 (−0.96,0.22) (−1.37,0.65)	−1.29 (−2.09,-0.57) (−2.62,-0.06)	−1.77 (−2.79,-0.69) (−3.56,0.22)
2017	−0.02 (−0.08,0.04) (−0.13,0.08)	−0.03 (−0.13,0.02) (−0.21,0.05)	−0.46 (−0.59,-0.34) (−0.68,-0.27)	−0.35 (−0.98,0.28) (−1.41,0.75)	−0.77 (−1.53,-0.07) (−2.15,0.46)	−1.06 (−2.08,0.07) (−2.88,0.93)
2018	−0.00 (−0.08,0.07) (−0.14,0.11)	−0.11 (−0.19,-0.05) (−0.25,-0.01)	−0.61 (−0.72,-0.49) (−0.80,-0.42)	−0.52 (−1.20,0.18) (−1.64,0.60)	−1.65 (−2.45,-0.99) (−3.03,-0.59)	−2.14 (−3.15,-0.98) (−3.96,-0.05)
2019	0.26 (0.16,0.36) (0.09,0.42)	−0.07 (−0.13,-0.01) (−0.19,0.02)	−0.31 (−0.44,-0.19) (−0.53,-0.12)	0.47 (−0.08,1.04)(−0.42,1.43)	−0.28 (−1.05,0.42) (−1.60,0.87)	0.28 (−0.73,1.40) (−1.55,2.26)
2020	0.29 (0.17,0.42) (0.09,0.50)	−0.04 (−0.10,0.00) (−0.15,0.03)	−0.25 (−0.37,-0.14) (−0.45,-0.07)	0.48 (−0.10,1.06) (−0.47,1.44)	0.34 (−0.24,0.94) (−0.62,1.36)	0.87 (−0.14,1.92) (−0.89,2.76)
2021	0.24 (0.15,0.32) (0.09,0.37)	−0.07 (−0.14,-0.01) (−0.20,0.01)	−0.37 (−0.52,-0.24) (−0.61,-0.17)	−0.16 (−0.66,0.34) (−0.96,0.69)	0.00 (−0.60,0.61) (−1.01,1.05)	−0.18 (−1.12,0.88) (−1.88,1.76)
2022	0.00 (−0.08,0.07) (−0.14,0.13)	−0.13 (−0.20,-0.05) (−0.25,0.00)	−0.43 (−0.53,-0.33) (−0.59,-0.28)	−0.49 (−1.42,0.23) (−1.87,0.66)	−1.21 (−2.02,-0.36) (−2.55,0.11)	−1.68 (−2.44,-0.82) (−3.05,-0.04)
Wct	MO			ER		
	Dir	Ind	Tot	Dir	Ind	Tot
2015	0.06 (0.01,0.12) (−0.02,0.15)	0.02 (−0.03,0.06) (−0.05,0.08)	0.07 (0.05,0.10) (0.03,0.12)	0.19 (0.14,0.24) (0.10,0.28)	0.01 (−0.03,0.06) (−0.07,0.11)	0.20 (0.18,0.23) (0.17,0.25)
2016	−0.13 (−0.19,-0.08) (−0.22,-0.04)	−0.02 (−0.06,0.02) (−0.09,0.06)	−0.15 (−0.19,-0.11) (−0.21,-0.08)	−0.00 (−0.04,0.04) (−0.06,0.07)	0.02 (−0.01,0.06) (−0.03,0.09)	0.02 (−0.01,0.06) (−0.03,0.08)
2017	−0.13 (−0.20,-0.07) (−0.23,-0.04)	−0.03 (−0.06,0.01) (−0.09,0.04)	−0.16 (−0.22,-0.09) (−0.26,-0.05)	−0.02 (−0.08,0.04) (−0.13,0.08)	0.05 (−0.00,0.11) (−0.03,0.16)	0.03 (−0.05,0.13) (−0.12,0.19)
2018	−0.19 (−0.27,-0.09) (−0.33,-0.03)	−0.12 (−0.18,-0.04) (−0.23,0.02)	−0.30 (−0.38,-0.20) (−0.42,-0.14)	−0.00 (−0.08,0.07) (−0.14,0.11)	−0.01 (−0.08,0.08) (−0.13,0.14)	−0.02 (−0.12,0.10) (−0.19,0.18)
2019	0.07 (−0.00,0.15) (−0.04,0.20)	0.06 (−0.01,0.12) (−0.05,0.16)	0.13 (0.06,0.21) (0.01,0.27)	0.26 (0.16,0.36) (0.09,0.42)	0.40 (0.28,0.56) (0.21,0.69)	0.66 (0.53,0.83) (0.44,0.97)
2020	0.08 (−0.01,0.19) (−0.06,0.26)	0.09 (0.01,0.18) (−0.05,0.24)	0.18 (0.11,0.26) (0.06,0.31)	0.29 (0.17,0.42) (0.09,0.50)	0.57 (0.41,0.73) (0.31,0.85)	0.86 (0.71,1.02) (0.61,1.15)
2021	−0.00 (−0.07,0.07) (−0.12,0.12)	0.05 (0.01,0.10) (−0.02,0.15)	0.05 (−0.02,0.13) (−0.07,0.18)	0.24 (0.15,0.32) (0.09,0.37)	0.44 (0.31,0.60) (0.24,0.75)	0.68 (0.52,0.87) (0.40,1.03)
2022	−0.16 (−0.23,-0.08) (−0.28,-0.01)	−0.04 (−0.11,0.03) (−0.16,0.09)	−0.20 (−0.24,-0.16) (−0.26,-0.13)	0.00 (−0.08,0.07) (−0.14,0.13)	0.02 (−0.05,0.09) (−0.10,0.15)	0.01 (−0.06,0.10) (−0.12,0.17)
	W-TP			Wct-TP		
	Dir	Ind	Tot	Dir	Ind	Tot
2015	−0.3 (−0.48,0.18) (−0.66,0.46)	−0.3 (−0.48,0.18) (−0.66,0.46)	−0.3 (−0.48,0.18) (−0.66,0.46)	0.02 (−0.26,0.38) (−0.47,0.53)	1.05 (−2.74,4.55) (−5.85,8.20)	1.16 (−2.84,4.71) (−6.14,8.46)
2016	−0.65 (−0.84,-0.15) (−0.94,0.19)	−0.65 (−0.84,-0.15) (−0.94,0.19)	−0.65 (−0.84,-0.15) (−0.94,0.19)	−0.37 (−0.53,-0.00) (−0.64,0.11)	0.53 (0.22,0.91) (0.07,1.16)	0.20 (−0.27,0.83) (−0.54,1.14)
2017	−0.61 (−0.78,-0.14) (−0.86,0.16)	−0.61 (−0.78,-0.14) (−0.86,0.16)	−0.61 (−0.78,-0.14) (−0.86,0.16)	−0.36 (−0.51,-0.03) (−0.59,0.10)	0.31 (0.17,0.51) (0.11,0.67)	−0.02 (−0.30,0.43) (−0.44,0.67)
2018	−0.26 (−0.32,-0.09) (−0.36,0.03)	−0.26 (−0.32,-0.09) (−0.36,0.03)	−0.26 (−0.32,-0.09)(−0.36,0.03)	−0.17 (−0.24,-0.04) (−0.28,0.01)	0.07 (0.02,0.13) (−0.01,0.18)	−0.10 (−0.21,0.07) (−0.27,0.17)
2019	−0.13 (−0.18,-0.00) (−0.20,0.08)	−0.13 (−0.18,-0.00) (−0.20,0.08)	−0.13 (−0.18,-0.00) (−0.20,0.08)	−0.05 (−0.11,0.02) (−0.13,0.06)	0.10 (0.06,0.16) (0.04,0.20)	0.04 (−0.04,0.17) (−0.08,0.25)
2020	−0.29 (−0.39,-0.04) (−0.45,0.11)	−0.29 (−0.39,-0.04) (−0.45,0.11)	−0.29 (−0.39,-0.04) (−0.45,0.11)	−0.14 (−0.26,0.01) (−0.32,0.09)	0.18 (0.10,0.31) (0.05,0.42)	0.04 (−0.14,0.31) (−0.25,0.48)
2021	−0.39 (−0.53,-0.07) (−0.60,0.12)	−0.39 (−0.53,-0.07) (−0.60,0.12)	−0.39 (−0.53,-0.07) (−0.60,0.12)	−0.20 (−0.36,-0.02 )(−0.44,0.08)	0.33 (0.21,0.47) (0.13,0.60)	0.15 (−0.11,0.40) (−0.28,0.58)
2022	−0.38 (−0.52,-0.11) (−0.59,0.06)	−0.38 (−0.52,-0.11 )(−0.59,0.06)	−0.38 (−0.52,-0.11) (−0.59,0.06)	−0.25 (−0.38,-0.10) (−0.46,-0.01)	0.08 (−0.05,0.20) (−0.15,0.30)	−0.17 (−0.39,0.07) (−0.57,0.21)

**Table 4:** Mean impacts.

	W			Wct		
	Mean Ind	Mean Tot	Mean Dir	Mean Ind	Mean Tot	Mean Dir
EPU	−0.06	−0.44	−0.39	−0.06	−0.44	−0.39
FP	−0.55	−0.64	−0.09	−0.55	−0.64	−0.09
MO	0.00	−0.05	−0.05	0.00	−0.05	−0.05
ER	0.18	0.33	0.15	0.18	0.33	0.15
TP	0.11	−0.27	−0.38	0.11	−0.27	−0.38
Ave	−0.06	−0.21	−0.15	−0.06	−0.21	−0.15

Table 3 provides insights into the direct, indirect, and total impacts of different policy uncertainty factors on industry returns, as well as their relative importance through the indirect-to-total effect ratio (Ind/Tot). The sign of the Mean Tot determines whether a policy uncertainty factor exerts an overall positive or negative impact on industry returns on average. A positive mean total impact suggests that policy uncertainty may create opportunities or stimulate industry growth (e.g., increased government spending under fiscal uncertainty), while a negative total impact implies that uncertainty imposes constraints on industry performance, possibly through reduced investment or higher risk premiums. Similarly, the sign of direct and indirect effects provides additional insights into the transmission mechanism. A positive Mean Ind indicates that spillovers reinforce the direct effects, leading to network amplification, whereas a negative indirect effect suggests that policy-induced disruptions in one industry may benefit others, reflecting competitive or substitution effects.

The Ind/Tot ratio further quantifies the relative importance of indirect effects in explaining the total impact of policy uncertainty. A positive Ind/Tot ratio implies that indirect spillovers move in the same direction as the direct effects, meaning that policy uncertainty propagates across industries in a reinforcing manner. In contrast, a negative Ind/Tot ratio suggests that indirect spillovers offset the direct impact, likely due to competitive shifts between industries. For instance, trade policy uncertainty (TP) has an Ind/Tot ratio of −0.39, indicating that while some industries experience direct negative effects (e.g., export-oriented sectors facing higher trade barriers), others benefit indirectly (e.g., domestic industries gaining market share). This dynamic suggests that trade uncertainty can lead to industry realignment rather than uniform contraction. The average absolute value of the Ind/Tot ratios across the five groups is 0.39, indicating that the network effects account for approximately 39% of the response of A-share industry returns to policy uncertainty on average.

Table 3 also highlights that fiscal policy uncertainty (FP) exhibits the strongest spillover effects, with an Ind/Tot ratio of 0.85, indicating that the majority of its impact is transmitted through network spillovers rather than direct influences. Similarly, exchange rate uncertainty (ER) has an Ind/Tot ratio of 0.55, suggesting that its effects extend beyond directly affected industries, likely through trade and currency exposure channels. In contrast, monetary policy uncertainty (MO) has an almost negligible effect, with an Ind/Tot ratio of 0.02, indicating that monetary uncertainty does not generate significant spillovers, possibly due to market expectations absorbing policy adjustments. A comparison between time-varying (W) and constant (Wct) spatial weight matrices shows that network effect structures remain stable, with minor differences in magnitude.

The observed average absolute Ind/Tot ratio of 0.23 indicates that direct impacts constitute the dominant transmission channel, while network spillovers contribute approximately 23% of total policy uncertainty effects. However, the presence of negative Ind/Tot ratios suggests that conventional arithmetic mean calculations may underestimate the actual spillover magnitude. Our analysis therefore employs absolute-value weighted averaging to more precisely quantify cross-sector transmission intensity. These results provide empirical evidence supporting the theoretical distinction between direct and network-mediated policy effects [24], with important implications for: risk assessment methodologies in interconnected markets, and policy design considering sectoral vulnerability to network spillovers. The findings particularly highlight the conditional nature of transmission mechanisms, where market volatility and policy uncertainty levels significantly modulate effect sizes.

## 4. Conclusions and recommendations

### 4.1. Conclusions

Regarding $\beta_{t}$ and $\alpha_{t}$, the analysis reveals significant volatility under EPU, FP, and MO, reflecting broad policy impacts, while ER shows heterogeneous patterns, with Transportation Equipment (N7) most sensitive to exchange rate fluctuations. TP results in lower beta volatility, indicating more stable sectoral responses. $\alpha_{t}$ analysis highlights FP, MO, and ER as major drivers of profitability shifts, with Resident Services (N15) under TP and Education (N16) under FP showing extreme sensitivity. Comparing time-varying and fixed weight matrices, the former captures amplified beta fluctuations but minimal differences in alpha values, suggesting adaptive market responses to dynamic linkages while maintaining stable long-term profitability.

Regarding systemic risk factors, the trends show a gradual upward trajectory under both time-varying and invariant weight restructuring, with mean values reflecting economic events. The EPU and FP groups exhibit surges followed by smoother fluctuations, while MO and ER groups show slow increases and declines, and the TP group decreases steadily. Time-varying weights capture greater heterogeneity across industries, evidenced by wider confidence intervals, whereas invariant weights reflect average systemic risk over time [25].

Regarding production network, this research reveals the directional flow of uncertainty across industries, highlighting China’s economic transition from industry-led to a balanced industrial-service structure. In the first half of the period, manufacturing (N3), electricity, heat, gas, and water production (N4), and mining (N2) dominate uncertainty transmission, while service sectors like culture, sports, and entertainment (N18) and financial services (N10) also play significant roles. In the second half, leasing and business services (N12) and construction (N5) emerge as key recipients and transmitters of uncertainty, reflecting their growing importance. The network underscores the foundational role of manufacturing and the rising influence of service industries, providing insights for optimizing industrial policies and fostering high-quality development [11].

Regarding spillover effect, this study reveals the direct, indirect, and total impacts of policy uncertainty on industry returns, with the indirect-to-total effect ratio (Ind/Tot) quantifying the relative importance of spillovers. Positive total impacts suggest uncertainty may stimulate growth, while negative impacts indicate constraints. The Ind/Tot ratio highlights whether spillovers reinforce (positive) or offset (negative) direct effects. For instance, trade policy uncertainty (TP) shows a negative Ind/Tot ratio (−0.39), reflecting industry realignment, while fiscal policy uncertainty (FP) exhibits strong spillovers with ratio value 0.85. Exchange rate uncertainty (ER) also shows significant indirect effects with ratio value 0.55, whereas monetary policy uncertainty (MO) has minimal spillovers with ratio value 0.02. Direct impacts dominate overall, with network effects accounting for about one-fourth of the total impact. These findings underscore the importance of distinguishing between direct and network spillover effects in policy assessments under varying uncertainty and market conditions.

### 4.2. Recommendations

Based on the findings, the following policy recommendations are proposed to mitigate the adverse effects of policy uncertainty and promote economic stability and growth:

Sector-Specific Support Mechanisms: Industries such as Transportation Equipment (N7) and Resident Services (N15) exhibit high sensitivity to specific uncertainties. Policymakers should design sector-specific support mechanisms, such as subsidies, tax incentives, or trade facilitation programs, to mitigate the impacts of exchange rate and trade policy uncertainties on vulnerable sectors [26].

Enhancing Resilience in Education and Public Services: The Education sector (N16) is particularly vulnerable to fiscal policy uncertainty. Governments should prioritize stable funding for education and public services to ensure their resilience and continuity, even during periods of budgetary uncertainty.

Promoting Industrial Transition: The sustainable production network highlights the growing importance of service industries like leasing and business services (N12) and construction (N5) in China’s economic transition. Policymakers should support this shift by investing in infrastructure, skills development, and innovation in these sectors while maintaining the foundational role of manufacturing.

Monitoring and Managing Network Effects: Policymakers should recognize the significant role of indirect spillovers, particularly under fiscal and exchange rate uncertainties. Comprehensive monitoring and management of these network effects can help mitigate unintended consequences and amplify positive outcomes.

Enhancing Stability in Core Social Sectors: The analysis reveals the critical vulnerability of education and public services to fiscal policy uncertainty. Implementing institutional safeguards like multi-year budget commitments and dedicated stabilization funds would ensure service continuity and strengthen these essential sectors during economic fluctuations.

Systemic Risk Monitoring Through Network Analysis: The results highlight the necessity of comprehensive monitoring systems that account for production network effects. Developing integrated early warning mechanisms combining real-time sectoral tracking with network analysis could better anticipate and mitigate cascading impacts, particularly from fiscal and exchange rate policy changes.

By implementing these recommendations, policymakers can better manage the impacts of policy uncertainty, foster economic stability, and support sustainable growth across industries.

## Appendix A

**Table A1:** Sample industies.

N1	Agriculture, forestry, animal husbandry and fisheries
N2	Mining
N3	Manufacturing
N4	Electricity, heat, gas and water production and supply
N5	Construction
N6	Wholesale and retail trade
N7	Transportation, storage and postal services
N8	Accommodation and catering
N9	Information transmission, software and information technology services
N10	Finance
N11	Real Estate
N12	Leasing and Business Services
N13	Scientific Research and Technology Services
N14	Water Resources, Environment and Public Facilities Management
N15	Residential Services, Repair and Other Services
N16	Education
N17	Health and social work
N18	Culture, Sports and Recreation

## Supporting information

S1 File Data. (ZIP)
